# Association Between Smoking and Hypertension in Pregnancy Among Japanese Women: A Meta-analysis of Birth Cohort Studies in the Japan Birth Cohort Consortium (JBiCC) and JECS

**DOI:** 10.2188/jea.JE20220076

**Published:** 2023-10-05

**Authors:** Naho Morisaki, Taku Obara, Aurelie Piedvache, Sumitaka Kobayashi, Chihiro Miyashita, Tomoko Nishimura, Mami Ishikuro, Fumihiro Sata, Reiko Horikawa, Chisato Mori, Hirohito Metoki, Kenji J Tsuchiya, Shinichi Kuriyama, Reiko Kishi

**Affiliations:** 1 Department of Social Medicine, National Center for Child Health and Development, Tokyo, Japan; 2 Tohoku Medical Megabank Organization, Tohoku University, Sendai, Japan; 3 Center for Environmental and Health Sciences, Hokkaido University, Sapporo, Japan; 4 Center for Child Mental Development, Hamamatsu University School of Medicine, Hamamatsu, Japan; 5 Health Center, Chuo University, Tokyo, Japan; 6 Division of Endocrinology and Metabolism, National Center for Child Health and Development, Tokyo, Japan; 7 Department of Bioenvironmental Medicine, Graduate School of Medicine, Chiba University, Chiba, Japan; 8 Division of Public Health, Hygiene and Epidemiology, Tohoku Medical and Pharmaceutical University Faculty of Medicine, Sendai, Japan

**Keywords:** smoking, hypertensive disorders of pregnancy, preeclampsia, meta-analysis, birth cohort

## Abstract

**Background:**

Recent literature suggest the effect of maternal smoking on risk of hypertensive disorders in pregnancy (HDP) and preeclampsia may differ by ethnicity; however, studies on Asians are limited.

**Methods:**

We investigated the association of maternal smoking with HDP and preeclampsia using a common analysis protocol to analyze the association in six birth cohorts participating in a Japanese consortium of birth cohorts (JBiCC). Results were compared with-published results from cohorts not included in this consortium, and, where possible, we produced a meta-analysis including these studies.

**Results:**

Meta-analysis of four cohort studies including 28,219 participants produced an odds ratio (OR) of 1.24 (95% confidence interval [CI], 0.88–1.87) for the effect of smoking beyond early pregnancy compared to women who did not smoke during pregnancy. These results combined with those from the Japan Environment and Children’s Study (JECS) yielded an OR of 1.19 (95% CI, 1.00–1.43, *P* = 0.056). Meta-analysis results for categories of smoking volume were insignificant, but when combined with JECS yielded an OR of 0.86 (95% CI, 0.65–1.12) for smoking 1–4 cigarettes, 1.25 (95% CI, 0.98–1.60) for smoking 5–9 cigarettes, and 1.27 (95% CI, 1.04–1.54) for smoking 10 or more cigarettes per day. All effects were insignificant for preeclampsia.

**Conclusion:**

Our results suggest that the protective effects of smoking longer and smoking more on HDP and preeclampsia repeatedly observed among Europeans and North Americans likely do not hold for the Japanese.

## INTRODUCTION

Smoking is known to have an adverse effect on various health prognosis, and affects obstetric outcomes, such as stillbirth,^1^ preterm birth,^2^ and low birth weight^3^ during pregnancy. Conversely, the effect of smoking on hypertension during pregnancy is still unclear, with recent studies suggesting the effect differs by ethnicity. A large United States Natality Data analysis stratified by ethnicity showed that smoking increased risk of pregnancy induced hypertension (PIH) only among Asian/Pacific Islanders, while it reduced risk among Whites, Blacks, and Native Americans.^4^ A report from Hawaii, where 70% of the sample were Asian, also used Natality Data and reported smoking during pregnancy increased risk of preeclampsia,^5^ defined as hypertension accompanied with proteinuria developing after 20 weeks of pregnancy.^6^ While such differences by ethnicity are becoming more obvious, research on the effect of smoking among Asians is still very scarce.

A recent systematic review and meta-analysis that estimated the relationship between smoking during pregnancy and hypertensive disorders in pregnancy (HDP) stratified by ethnicity^7^ showed that smoking was a protective factor in Europe (odds ratio [OR] 0.67; 95% confidence interval [CI], 0.57–0.79) and North America (OR 0.78; 95% CI, 0.65–0.93) but a significant risk factor for HDP in Asia (OR 1.13; 95% CI, 1.03–1.24). However, only two studies^8^^,^^9^ were used for the Asian sub-analysis, and both were from Japan. Furthermore, as neither study had results on preeclampsia, and only one study could identify women who quit smoking during pregnancy, such sub-analyses were left unconducted for the Asian population.

In 2019, the Japanese birth cohort consortium (JBiCC) to study the relationship between early life environment and maternal and child outcomes was launched to aggregate information from various birth cohorts in Japan to understand and overcome common pregnancy complications, including HDP. Thus, using this platform, we aimed to investigate the effect of smoking on HDP and preeclampsia in Japan. However, while our consortium includes two of the largest birth cohorts in Japan, there are numerous cohorts that are not part of JBiCC as of April 2022. Thus, to provide a comprehensive understanding of this subject among Japanese, we took a two-step approach of first conducting meta-analysis of aggregated results obtained from each cohort using a common analysis protocol, and second, identifying published results from cohorts not part of JBiCC but had utilized a comparable analysis protocol and conducting a meta-analysis including these results.

## METHODS

### Study population

This study was based on an on-going collaboration platform among birth cohort studies in Japan that was launched in 2019. At launch, six cohort studies participated in the collaboration platform. The Hokkaido Study on Environment and Children’s Health recruited pregnant women and their fetuses from 2002. It consists of two cohorts, the Sapporo cohort (*n* = 514) and the Hokkaido large-scale cohort (*n* = 20,940).^10^ The Tohoku Medical Megabank Project Birth and Three-Generation Cohort Study (TMM BirThree Cohort Study) started in 2013, and recruited 22,493 pregnant women in Miyagi Prefecture.^11^^,^^12^ The Babies and their Parents’ Longitudinal Observation in Suzuki Memorial Hospital in Intrauterine Period (BOSHI) study recruited 1,576 pregnant women from 2006 in a hospital specializing in obstetrics, gynecology and in-vitro fertilization in Miyagi Prefecture.^13^ Chiba study of Mother and Children’s Health (C-MACH) recruited 433 pregnant women at three hospitals and clinics.^14^ Babies’ fathers were also recruited. The Seiiku Boshi Birth Cohort is a hospital-based birth cohort study starting in 2010, and 2,310 pregnant women participated in the study.^15^ The Hamamatsu Birth Cohort for Mothers and Children (HBC Study) recruited 1,138 pregnant women at a hospital and a clinic from 2007.^16^

Upon initiation of this study, each cohort provided numbers of women who had HDP in their cohorts. To ensure the analyses conducted at the individual cohort level had sufficient sample size to adjust for important confounders, we limited the analysis to cohorts which had at least 100 cases. Thus, the following four cohorts were included: the Hokkaido large-scale cohort, the TMM BirThree Cohort Study, the BOSHI study, and the HBC Study. Among the participating cohorts, pregnant women who had multiple pregnancies, pregnancies that ended in miscarriage/abortion/still birth, or who had histories of HDP or gestational diabetes mellitus were excluded from the study population.

### Outcomes

HDP and preeclampsia were collected from medical records and defined using either raw clinical data on blood pressure and proteinuria or doctor’s diagnosis at time of data collection. Each cohort used the closest definition to that in the current clinical guideline of HDP and preeclampsia^17^ from the data available in each cohort. The exact definition is shown in eTable 1. The definition slightly differed by cohort, as BOSHI defined HDP as hypertension during pregnancy (systolic blood pressure above 140 mm Hg or diastolic blood pressure above 90 mm Hg occurring after 20 weeks of gestation and dissolving within 12 weeks after delivery) observed at least twice, while TMM BirThree considered cases where hypertension was observed only once also as HDP. The definition of proteinuria for preeclampsia also slightly differed, as TMM BirThree cohort considered one positive test of protein ≥2+ dipstick as proteinuria, while BOSHI considered cases with multiple observations of dipstick proteinurea of 1+, or more than one occurrence of dipstick proteinurea of 2+, proteinurea ≥27 mg/g Cre, or proteinurea ≥30 mg/dL as HDP. Hokkaido and Hamamatsu did not have detailed data on blood pressure and urine measurements, and clinical diagnoses were used. For subjects who delivered in 2003–2004 in the Hokkaido Cohort, only information of “toxemia in pregnancy” defined as those who had edema in addition to hypertension and proteinuria^18^ was available; thus, women with “toxemia in pregnancy” were labeled having HDP as well as preeclampsia. The HBC study did not have information on preeclampsia, so their results were used only for analyses regarding HDP.

### Smoking and covariates

Smoking status was collected from self-reported questionnaires delivered in each cohort. As the questions used to assess smoking differed by cohort, consensus on categorization on smoking status was reached through discussion. The reached consensus for categorization as well as how they correspond to the original questions asked in each cohort are shown in eTable 2.

First, smoking status was categorized as “no smoking during pregnancy (never smoker or quit smoking before week 0 of pregnancy)”, and “Smoking during early pregnancy”. “Smoking during early pregnancy” was further categorized categorized into “quit smoking during early pregnancy (0–11 weeks of gestation)” and “continued smoking beyond early pregnancy (beyond 12 weeks of gestation)”. Finally, the number of cigarettes smoked during early pregnancy were categorized as “1–4”, “5–9” and “10 or above”. Data on number of cigarettes were not available in HBC study.

Covariates were selected and adjusted in the analysis by each cohort study from the following variables: maternal age at childbirth, history of previous pregnancies, pre-pregnancy body mass index (BMI), socioeconomic status (income, educational attainment or job status), passive smoking, alcohol consumption, and child sex.

### Identification of studies not involved in JBiCC

To provide a comprehensive understanding of the effect of maternal smoking on risk of HDP on pre-eclampsia, we conducted a literature review to identify previous studies regarding this subject conducted among Japanese. As a recent systematic review^7^ on relevant studies up to March 2021 had identified two Japanese studies from Japan, we used the same search terms and inclusion and exclusion criteria to identify studies that had been published from April 2021 to April 2022. The initial search yielded 121 results, and title and abstract screening by two authors identified one study on smoking (use of heated tobacco product use) and risk of HDP in Japanese.

Through this search three studies were identified for possible inclusion: one cohort study by Tanaka using the Japan Environmental Children’ Study (JECS),^8^ one cross-sectional study by Hayashi using the Japan Perinatal Registry Network database,^9^ and one cross-sectional study by Zaitsu using the Japan COVID-19 and Society Internet Survey (JACSIS) study.^19^ Of these three studies, the study by Hayashi only reported the effect of overall maternal smoking (not limited to early pregnancy) and the study by Zaitsu only reported the effect of heated tobacco products, so these were considered as having an analysis protocol where results were not combinable with those from JBiCC. Thus, in the end only the Tanaka study which utilized JECS was included.

### Statistical analyses

Three analyses were performed on effect of smoking on risk of HDP and preeclampsia: 1) the effect of smoking during early pregnancy, compared to no smoking during pregnancy; 2) the effect of quitting smoking between 0–11 weeks, as well as continuous smoking beyond early pregnancy, compared to no smoking during pregnancy; and 3) the effect of smoking 1–4 cigarettes per day, 5–9 cigarettes per day, or 10 or more cigarettes per day between 0 and 11 gestational weeks, compared to 0 cigarettes per day.

For each analysis, we used a two-step approach. In the first step, each cohort analyzed their data independently by using multiple logistic regression to investigate the association between smoking and HDP/PE adjusted for covariates. Software used were STATA (version 14.0 or 16.0; StataCorp LP, College Station, TX, USA), SAS (version 9.4; SAS Institute Inc., Cary, NC, USA) or SPSS version 26 (IBM Corp., Armonk, NY, USA).

The second step for the analysis was meta-analyses with common-effect inverse-variance models using the aggregated results from the first step. We compared our results with previous reports from Japan that also provided ORs for the association between smoking and risk of HDP/preeclampsia, and, where possible, produced a meta-analysis including these studies. Heterogeneity between individual studies were assessed graphically and with the I^2^ statistic. A random-effects model with bootstrapped DerSimonian-Laird method was performed in comparison when a heterogeneity over 20% was detected. Sensitive analysis simulating a heterogeneity of 80% was performed to assess undetected heterogeneity due to small number of studies.^20^ Additionally, we conducted influence analysis by repeating analysis on HDP omitting one cohort at a time. This was not conducted for PE as the meta-analysis was on only three studies. All analyses in the second step were performed using STATA (version 14.0).

### Ethical considerations

Written informed consent was obtained from all participants of each of the four cohorts. Based on this, analysis was conducted within each cohort by researchers who were allowed to have access to individual data. Aggregated data was sent to the National Center of Child Health Development where the meta-analysis was conducted. The study protocol was approved by the Ethical Committee of the National Center of Child Health Development on April 5, 2021 (approval number 2021-004).

## RESULTS

In Table 1 we show summary statistics of the population that was included in the analyses. Of the four cohorts that participated in the meta-analysis, the number of subjects largely differed with the two larger cohorts having 16,733 subjects in Hokkaido cohort and 8,979 subjects in TMM BirThree cohort, and the two smaller cohorts having 1,258 subjects in the HBC cohort and 1,249 subjects in the BOSHI cohort. Prevalence of HDP was very low in the Hokkaido cohort (1.2%) compared to the other cohorts, where prevalence ranged from 10.2% to 11.4%. Prevalence of PE ranged from 1.0% to 2.5%.

**Table 1.** Basic characteristics of participating cohorts

	Hokkaido Cohort (*n* = 16,733)		TMM BirThree Cohort (*n* = 8,979)		HBC Study (*n* = 1,258)		BOSHI Cohort (*n* = 1,249)	
Outcomes	*n*	%	*n*	%	*n*	%	*n*	%
Hypertensive disorders in pregnancy	201	1.2%	920	10.2%	143	11.4%	137	11.0%
Preeclampsia	164	1.0%	226	2.5%	No information		24	1.9%
Smoking status								
No smoking during pregnancy	10,581	63.2%	8,118	90.4%	989	78.6%	1,035	82.9%
Smoking during early pregnancy	6,152	36.8%	861	9.6%	269	21.4%	214	17.1%
Quit smoking during early pregnancy (0–13 weeks)	6,051	36.2%	617	6.9%	203	16.1%	166	13.3%
Continued smoking beyond early pregnancy	101	0.6%	244	2.7%	66	5.2%	48	3.8%

Volume of smoking during early pregnancy								
No smoking	10,581	63.2%	8,118	90.4%	989		1,035	82.9%
1 to 4 cigarettes per day	475	2.8%	246	2.7%	No information		9	0.7%
5 to 9 cigarettes per day	666	4.0%	92	1.0%	No information		39	3.1%
10 or more cigarettes per day	852	5.1%	523	5.8%	No information		166	13.3%
Smoked but volume unknown	4,159	24.9%	0	0.0%	No information		0	0.0%

	Hokkaido Cohort (*n* = 16,733)		TMM BirThree Cohort (*n* = 8,979)		HBC Study (*n* = 1,258)		BOSHI Cohort (*n* = 1,249)	
Background Characteristics	mean	SD	mean	SD	mean	SD	mean	SD
Maternal age, years	30.3	4.8	32.0	4.8	31.5	5.1	31.2	4.9
Pre-pregnancy body mass index, kg/m^2^	21.1	3.3	21.3	3.3	21.0	3.3	21.7	3.4
Gestational weight gain, kg	No information		10.1	4.2	10.5	4.4	10.1	4.2
Gestational age at delivery, weeks	38.8	1.5	38.8	1.6	38.9	1.6	39.6	1.7
	*n*	%	*N*	%	*n*	%	*n*	%
Primiparity (no previous delivery)	7,093	42.4	2,940	32.7	626	49.8	717	57.4
Infant sex male	9,284	55.4	4,666	52.0	647	51.4	637	51.0

^a^TMM BirThree: Tohoku Medical Megabank Project Birth and Three-Generation Cohort Study.

^b^HBC Study: Hamamatsu Birth Cohort for Mothers and Children Study.

^c^BOSHI Cohort: Babies and their Parents’ Longitudinal Observation in Suzuki Memorial Hospital in Intrauterine Period Study.

The proportion of women who smoked during early pregnancy was highest in the Hokkaido cohort (36.8%) and lowest in the TMM BirThree cohort (9.6%); however, the proportion of women continuing to smoke beyond early pregnancy was the lowest in the Hokkaido cohort (0.6%) and highest in the HBC cohort (5.2%). Among all three cohorts that had data on volume of smoking, the majority of women who smoked had 5 or more cigarettes per day, and for the TMM BirThree cohort and BOSHI cohort the majority had 10 or more cigarettes per day.

### Association between smoking and HDP

#### Effect of smoking during early pregnancy compared to no smoking during pregnancy

**Figure 1.**
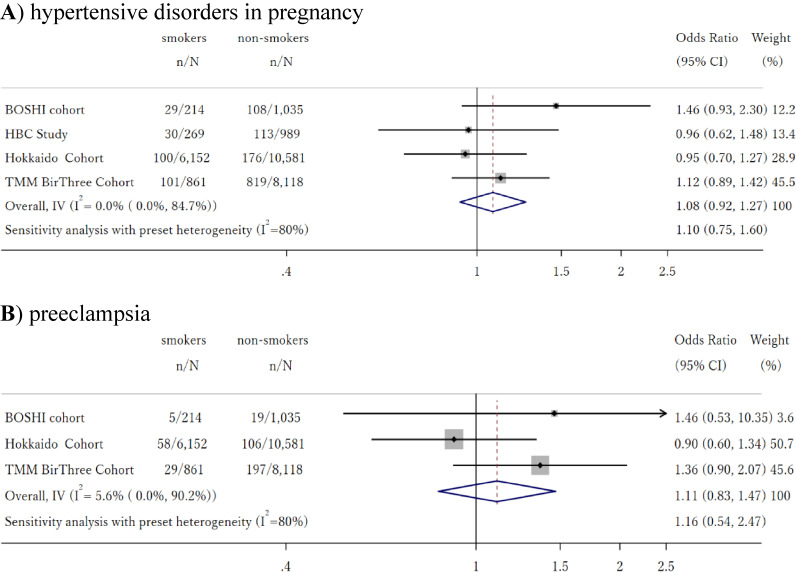
Association between smoking during early pregnancy and risk of hypertensive disorders in pregnancy and preeclampsia

In Figure 1A, we show the association between smoking in early pregnancy and risk of HDP. The associations within each individual cohort were insignificant in all cohorts and were homogeneous (I-squared = 0%). Meta-analysis pooling of ORs using the common-effect inverse-variance model produced an OR of 1.08 (95% CI, 0.93–1.27) for effect of smoking during early pregnancy, compared to no smoking during pregnancy. Sensitivity analysis expanding the definition of non-smoker to those who quit smoking shortly after they realized they were pregnant produced similar results, with OR of 1.02 (95% CI, 0.85–1.22) (eFigure 1A).

In Figure 1B we show the association between smoking in early pregnancy and risk of PE. The associations within each individual cohort were similar to those observed for HDP. Meta-analysis produced an OR of 1.11 (95% CI, 0.83–1.47) for effect of smoking during early pregnancy compared to no smoking during pregnancy. Sensitivity analysis expanding the definition of non-smoker to those who quit smoking shortly after they realized they were pregnant produced similar results, with an OR of 1.05 (95% CI, 0.78–1.47) (eFigure 1B).

#### Effect of smoking only during early pregnancy (quitting between 0–11 weeks), as well as continuous smoking beyond early pregnancy (after 12 weeks of gestation) compared to no smoking during pregnancy

**Figure 2.**
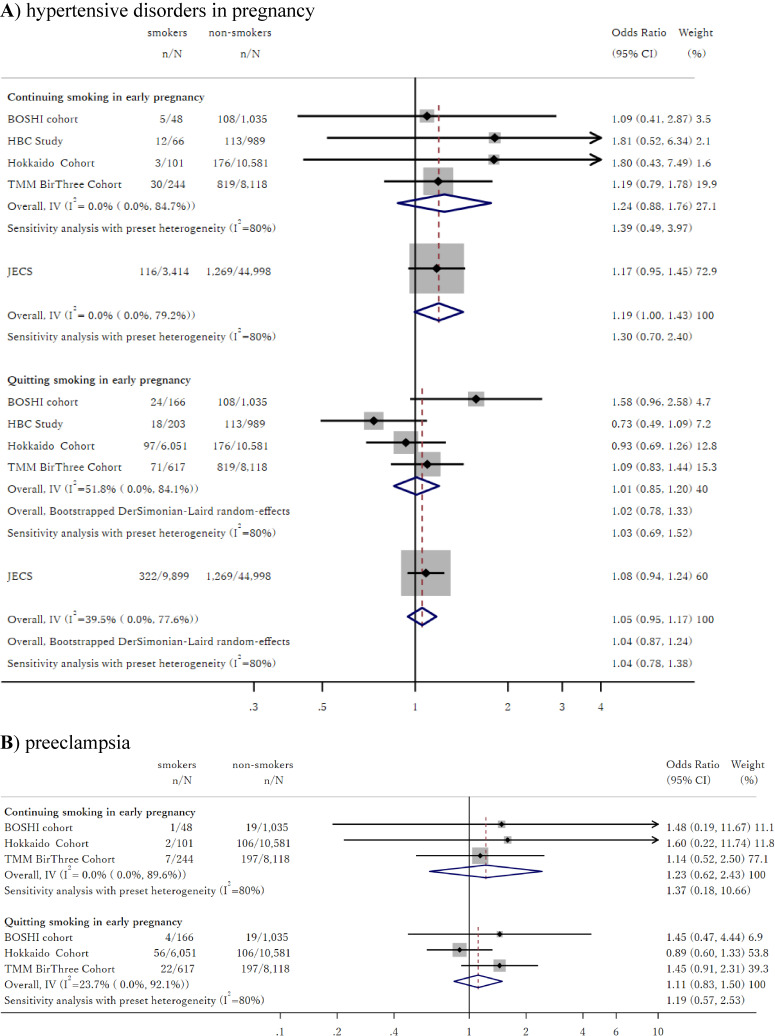
Association between smoking only during early pregnancy and continuing smoking beyond pregnancy, and risk of hypertensive disorders in pregnancy and PE

In Figure 2A, we show the association of smoking only during early pregnancy and beyond early pregnancy with risk of HDP compared to no smoking during pregnancy. The associations within each individual cohort were insignificant in all cohorts and showed heterogeneity for effect of smoking only during early pregnancy (I-squared = 52%) but not for continuous smoking beyond early pregnancy (I-squared = 0%). Meta-analysis pooling of ORs using the common-effect inverse-variance model produced non-significant ORs of 1.01 (95% CI, 0.86–1.20) for the effect of smoking only during early pregnancy and 1.24 (95% CI, 0.88–1.87) for the effect of smoking beyond early pregnancy.

These results did not show significant heterogeneity with results obtained from the JECS study (*P* = 0.78 for smoking only during early pregnancy, and *P* = 0.53 for smoking beyond early pregnancy). Meta-analysis including the JECS study yielded overall ORs of 1.05 (95% CI, 0.95–1.17) for the association between smoking only during early pregnancy and HDP and 1.19 (95% CI, 1.00–1.43) for the association between smoking beyond early pregnancy and HDP. Sensitivity analysis expanding the definition of non-smoker to those who quit smoking shortly after they realized they were pregnant produced similar results, with ORs of 0.96 (95% CI, 0.79–1.18) for the effect of smoking during early pregnancy, 1.23 (95% CI, 0.87–1.74) for the effect of smoking beyond early pregnancy among the four cohorts, and 1.04 (95% CI, 0.93–1.17) and 1.19 (95% CI, 0.99–1.43), respectively, when combined with the JECS study (eFigure 2A).

In Figure 2B, we show the association of smoking only during early pregnancy and beyond early pregnancy with risk of PE compared to no smoking during pregnancy. The point estimate of the OR differed from those for HDP in some cohorts; however, they were insignificant for all cohorts, similar to analyses for HDP. Meta-analysis produced ORs of 1.12 (95% CI, 0.83–1.50) for the effect of smoking during early pregnancy and 1.23 (95% CI, 0.62–2.44) for the effect of smoking beyond early pregnancy. Sensitivity analysis expanding the definition of non-smoker to those who quit smoking shortly after they realized they were pregnant produced similar results, with ORs of 1.04 (95% CI, 0.75–1.42) for the effect of smoking during early pregnancy and 1.21 (95% CI, 0.61–2.41) for the effect of smoking beyond early pregnancy (eFigure 2B).

#### Effect of smoking 1*–*4 cigarettes per day, 5–9 cigarettes per day, and 10 or more cigarettes per day, compared to 0 cigarettes per day, between 0 and 11 gestational weeks

**Figure 3.**
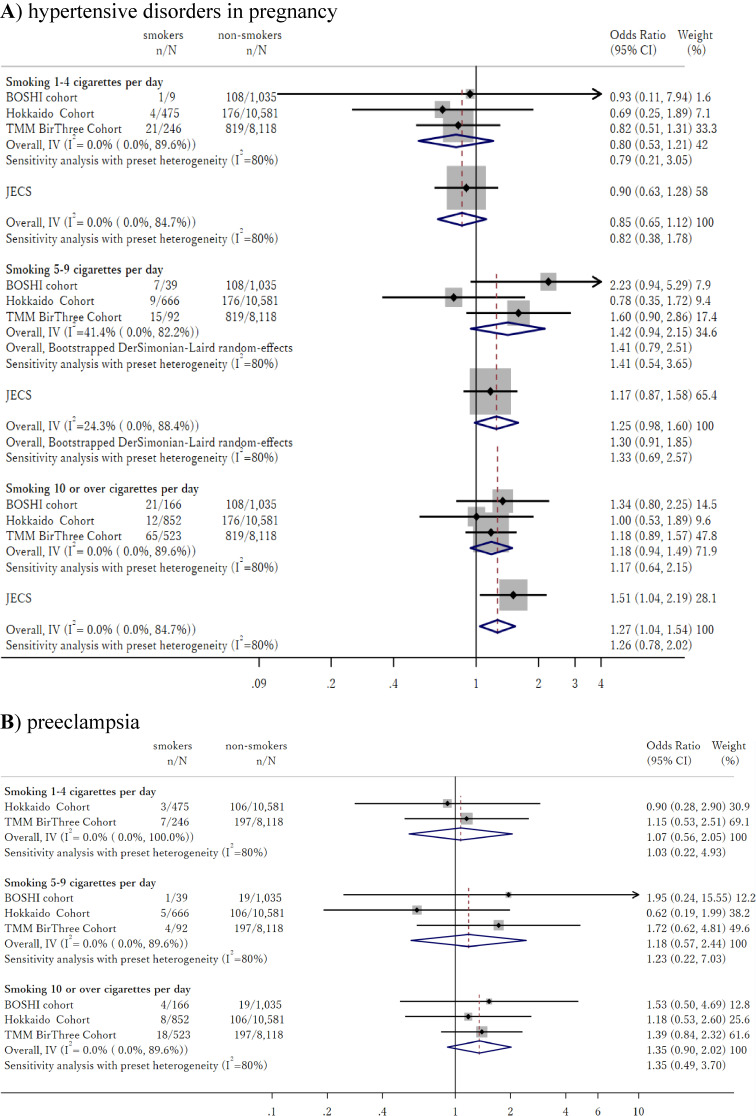
Association between volume of smoking during early pregnancy, and risk of hypertensive disorders in pregnancy and preeclampsia

In Figure 3A we show the association between number of cigarettes and risk of HDP. The association within each individual cohort were insignificant in all cohorts. Heterogeneity was observed for effect of smoking 5–9 cigarettes per day in early pregnancy (I-squared = 41%) but not for other categories (I-squared = 0%). Meta-analysis pooling of ORs using the common-effect inverse-variance model produced ORs of 0.80 (95% CI, 0.53–1.21) for smoking 1–4 cigarettes per day, 1.42 (95% CI, 0.94–2.15) for smoking 5–9 cigarettes per day, and 1.19 (95% CI, 0.94–1.49) for smoking 10 or more cigarettes per day.

These results did not show significant heterogeneity with results obtained from the JECS study (*P* = 0.68 for smoking 1–4 cigarettes per day, *P* = 0.46 for smoking 5–9 cigarettes per day, and *P* = 0.28 for smoking 10 or more cigarettes per day). Meta-analysis including the JECS study yielded overall ORs of 0.86 (95% CI, 0.65–1.12) for smoking 1–4 cigarettes per day, 1.25 (95% CI, 0.98–1.60) for smoking 5–9 cigarettes per day, and 1.27 (95% CI, 1.04–1.54) for smoking 10 or more cigarettes per day, compared to no smoking during early pregnancy.

Sensitivity analysis expanding the definition of non-smoker to those who quit smoking shortly after they realized they were pregnant produced similar results, with ORs of 0.82 (95% CI, 0.54–1.23) for smoking 1–4 cigarettes per day, 1.18 (95% CI, 0.75–1.86) for smoking 5–9 cigarettes per day, and 1.15 (95% CI, 0.89–1.49) for smoking 10 or more cigarettes per day for the three cohorts. After combining with the JECS study, we observed ORs of 0.86 (95% CI, 0.66–1.12) for smoking 1–4 cigarettes per day, 1.17 (95% CI, 0.91–1.51) for smoking 5–9 cigarettes per day, and 1.26 (95% CI, 1.02–1.55) for smoking 10 or more cigarettes per day (eFigure 3A).

In Figure 3B we show the association between number of cigarettes and risk of PE. The point estimate of the OR differed from those for HDP in some cohorts; however, they were insignificant for all cohorts, similar to analyses for HDP. Meta-analysis produced an OR of 1.07 (95% CI, 0.65–1.12) for smoking 1–4 cigarettes per day, 1.18 (95% CI, 0.57–2.44) for smoking 5–9 cigarettes per day, and 1.35 (95% CI, 0.90–2.02) for smoking 10 or more cigarettes per day. Sensitivity analysis expanding the definition of non-smoker to those who quit smoking shortly after they realized they were pregnant, produced similar results with OR of 1.06 (95% CI, 0.56–2.03) for smoking 1–4 cigarettes per day, 1.09 (95% CI, 0.51–2.36) for smoking 5–9 cigarettes per day, and 1.38 (95% CI, 0.91–2.11) for smoking 10 or more cigarettes per day. (eFigure 3B).

Influence analysis plots showing how meta-analysis results for HDP change when excluding each cohort are shown in eFigure 4. The direction of effect for increased risk in HDP for those who continued smoking past early pregnancy, smoked 5–9 cigarettes, and 10 or over cigarettes per day in early pregnancy, as well the direction of effect for decrease in risk in HDP for those who smoked 1–4 cigarettes per day, did not change when omitting any of the cohorts.

## DISCUSSION

We conducted a meta-analysis on the associations of smoking during pregnancy with HDP as well as preeclampsia among women in Japan using an uniformed analysis plan among cohorts participating in a newly created consortium (JBiCC) as well as a separately running large birth cohort (JECS). We observed that the association between smoking beyond early pregnancy and HDP was marginally insignificant (OR 1.19; 95% CI, 1.00–1.43; *P* = 0.056) and the association between smoking 10 or more cigarettes per day in early pregnancy and HDP was significant (OR 1.27; 95% CI, 1.04–1.54). All effects were insignificant for preeclampsia. As far as we know, this is the largest study observing the association of smoking with HDP and preeclampsia in the Japanese population. Our results failing to find a negative association between smoking and risk utilizing all information currently available in the Japanese suggest that the protective effects of smoking longer and smoking more on HDP and preeclampsia, which have been repeatedly observed among Europeans and North Americans, likely do not hold for the Japanese, and on the contrary, may possibly increase risk.

In our study we observed an OR of 1.24 (95% CI, 0.88–1.76) for risk of HDP and 1.23 (95% CI, 0.62–2.43) for risk of PE for those who continued smoking beyond early pregnancy compared to those who never smoked during pregnancy. The magnitude of the effect for HDP was similar to that reported in the JECS study,^8^ which was also insignificant (1.17; 95% CI, 0.95–1.45), and when combined provided a marginally insignificant effect (1.19; 95% CI, 0.995–1.43), with a *P*-value of 0.056. These estimates are similar to those reported for smoking during pregnancy and risk of PE in Hawaii^5^ (1.19; 95% CI, 1.07–1.33), as well as of PIH reported from Japan^9^ (1.20; 95% CI, 1.01–1.41). While we cannot deny the possibility of unadjusted confounding, it is likely that the true effect lies somewhere from mild to moderate (OR 1.0 to 1.5).

On the other hand, the association between smoking only during early pregnancy was less pronounced. The observed ORs were 1.01 (95% CI, 0.85–1.20) for HDP and 1.11 (95% CI, 0.83–1.50) for PE compared to those who never smoked during pregnancy. When combined with the JECS study, the OR was 1.05 (95% CI, 0.95–1.17). A recent meta-analysis on four studies among North Americans and Europeans also showed a non-significant relationship between smoking cessation before pregnancy and the incidence of HDP (OR 1.01; 95% CI, 0.94–1.08).^7^ This suggests, to some extent, that continued smoking during pregnancy may be an important condition for smoking to cause HDP or PE, and the effect of cigarettes may disappear after cessation of smoking.

While multiple studies and meta-analyses^21^^–^^23^ have confirmed a dose-response relationship, in which risk of preeclampsia decreases as the number of cigarettes smoked per day increases among the North American and European populations, our study, while all estimates were insignificant, showed the completely opposite relationship. OR point estimates increased as the number of cigarettes smoked per day increased (1.07; 95% CI, 0.65–1.12 for 1–4 cigarettes, 1.18; 95% CI, 0.57–2.44 for 5–9 cigarettes, and 1.35; 95% CI, 0.90–2.02 for 10 or more cigarettes). A similar association has been reported for HDP from the JECS study (OR 0.90; 95% CI, 0.63–1.28 for 1–4 cigarettes, OR 1.17; 95% CI, 0.87–1.58 for 5–9 cigarettes, and OR 1.51; 95% CI, 1.04–2.19 for 10 or more cigarettes), and when the findings from the four cohorts were combined with those from JECS, women smoking over 10 cigarettes per day showed a significant increase in risk in HDP (OR 1.26; 95% CI, 1.02–1.55).

The disproportionate association between smoking and HDP by ethnicity has only been recently acknowledged. Many systematic reviews and meta-analyses on studies from North America and Europe^21^^–^^23^ repeatedly have shown that, disturbingly, smoking reduces risk of HDP and preeclampsia. The protective effect of smoking on reducing blood pressure has even been confirmed through a large mendelian randomization meta-analysis.^24^ The underlying mechanism for this protective effect has been suggested to be due to the effect of nicotine, which inhibits thromboxane production,^25^ as well as dilates blood vessels,^26^ leading to lower blood pressure. However, at the same time, nicotine also hampers placental development both directly through inhibiting placental trophoblast cell invasion and indirectly by reducing blood flow,^27^ which can lead to increased risk of preeclampsia, as well as other HDP. This is thought to be the main mechanism for the increased risk of stillbirth^1^ and placental abruption^28^ among smokers, for which the adverse risk can be observed across populations. Thus, the total effect of smoking is not simple, and is rather a balance of its effect to increase blood pressure and its effect to reduce it, a balance which may differ by genetic differences among ethnicities.

As our study adds to the previous literature suggesting the adverse effect of smoking on HDP and PE among Japanese, further studies assessing why such ethic disparity on effect of smoking exist are needed. A previous study suggested that the traditional negative view towards female smoking may be leading to a certain bias in data collection.^7^ However, recent studies suggest at least two factors that may cause significantly modify the effect of smoking on HDP and PE, as mentioned below.

One possibility is that the effect of smoking may differ by maternal characteristics that are not genetic. A small case-control study in Poland, which showed that the adverse effect of smoking in the first trimester was disproportionately high among underweight women (OR 22) compared to other (normal, overweight, or obese) women,^29^ suggests the higher proportion of underweight among Japanese pregnant women may be the cause.

Another possibility is that the effect of smoking differs by inherited factors. For instance, recent evidence suggest genetic differences in speed of nicotine metabolism may play a role in explaining why smoking increases blood pressure selectively among Asians. A study in China reported that among CYP2A6 variations, the slow nicotine metabolism genotype, could be observed in 40% of the population, for whom smoking doubled the risk of hypertension while not influencing those with the normal metabolism genotype.^30^ This slow nicotine metabolizer genotype is rarely observed (allele frequency <5%) among Caucasians and much higher among Chinese (allele frequency = 15%) and is estimated to be even higher among Japanese (allele frequency = 20%).^31^ Further studies looking into whether there are interactions between smoking and genetic components, as well as other characteristics, are anticipated.

In our study, the proportion of women in the cohorts who were smoking at initiation of pregnancy ranged from 9% to 37%. We observed large regional differences that most likely reflect differences in smoking rates among women, which has been reported previously. Overall, proportions observed in our study were similar to those reported in JECS, where nearly 20% of women were smoking at beginning of pregnancy.^8^ On the other hand proportion of women who smoke during pregnancy was 0.6 to 5.2% in our survey. These figures were similar to those reported in a national survey (6.4%)^32^ and the JECS study (5.4%),^33^ with the exception of Hokkaido Cohort, where rate was very low (0.4%). The low rate in this cohort may be due to the structure of the questionnaire used, as well as social desirability bias leading to hesitation to answering continuation of smoking, resulting in a large proportion of women who answered they smoked at initiation of pregnancy not providing data on how much they smoked later in pregnancy (who were categorized as quitting smoking in early pregnancy). Social desirability bias may have influenced this decision of not providing information of smoking.

Incidence of HDP among the four cohorts in our study was 1.2% in the Hokkaido Cohort and 10–11% in the other three cohorts. As shown by Garovic et al, the incidence varies greatly depending on the population characteristics, and the value also varies depending on the reporting process.^26^ Previous studies in Japan have also shown large variability in prevalence of HDP (5.2–8.2%) and preeclampsia (0.2–9.2%).^34^ The higher prevalence observed in BirThree and BOSHI cohorts may also be reflecting the higher sensitivity of the criteria strictly based on blood pressure and proteinuria measurements at all antenatal care visits as well as delivery records, reducing the risk of underdiagnosis. On the other hand, the low prevalence in the Hokkaido cohort may be due to the cohort being older, and for those born in 2002–2004 only those who met the stricter criteria used to diagnose pregnancy toxemia were used as having HDP, resulting in the prevalence becoming similar to that of preeclampsia.

### Strengths and limitations

The strength of our study is that was based on a birth cohort collaboration platform in Japan. Thus, we were able to utilize a large overall population size and were able to evaluate not only the dichotomous effect of smoking but also how the association differed by duration of smoking or dose of smoking. In addition, as we conducted the analyses using a common analysis protocol and included all results regardless of significance, even though our study is a meta-analysis, it is free from publication bias. Heterogeneity was mostly small, and we further checked the robustness of our findings by conducting a sensitivity analysis to assess undetected heterogeneity due to small number of studies.^19^

However, our study has its limitations. First, even though we utilized data from four cohorts, two of which are the largest birth cohorts in Japan, our sample size was much smaller than previous meta-analyses conducted mainly among Caucasians, possibly leading to lack of power for some of the analyses. We tried to compensate for this limitation by utilizing published literature based on studies outside our consortium; however, as we could only identify one such study, future research from cohorts and other databases, based on a similar analysis protocol to the one we used, is encouraged. Second, while our results suggested a stronger relationship between smoking and HDP and PE among those who continued smoking beyond early pregnancy, and a stronger relationship among those who smoked a larger number of cigarettes during early pregnancy, we were not able to analyze on the effect of number of cigarettes in late pregnancy, as only one cohort collected such data. As one of the cohorts in our study has previously reported that higher rates of plasma cotinine level in the third trimester is associated with twice higher risk of HDP,^35^ future studies on the effect of smoking in late pregnancy are anticipated. Third, even though we adjusted for possible confounders, we cannot deny the possibility of unmeasured confounding. For instance, many of our cohorts did not have data on maternal blood pressure before pregnancy or previous medical history of HDP/preeclampsia. Finally, our results suggest that there are environmental or genetic factors that differ between the Japanese and North Americans/Europeans that modify the effect of smoking on HDP and PE, which also suggest that the effect among Japanese may differ by environmental or genetic differences as well; however, we were not able to conduct such stratified analyses within our population. More studies on why the effect of smoking differs between populations are needed.

### Conclusion

Our meta-analysis of birth cohorts in Japan demonstrated that the association between smoking past early pregnancy and HDP showed the same direction of increased risk in all the four cohorts and showed a marginally insignificant association when analyzed together with a previously published birth cohort (JECS). Our results suggest the protective effects of smoking longer and smoking more on HDP and preeclampsia repeatedly observed among Europeans and North Americans likely do not hold for the Japanese.

## SUPPLEMENTARY MATERIAL

eTable 1. Definitions of HDP and PE used in the cohorts

eTable 2. Definition of smoking and not smoking during pregnancy

eFigure 1. Association of smoking during early pregnancy with risk of HDP and PE

eFigure 2. Association of smoking only during early pregnancy and continuing smoking beyond pregnancy with risk of HDP and PE

eFigure 3. Association of volume of smoking during early pregnancy with risk of HDP and PE

eFigure 4. Influence analysis plot: meta-analysis estimates, given named study is omitted
