# Supplementary material for: Association Between Smoking and Hypertension in Pregnancy Among Japanese Women: A Meta-analysis of Birth Cohort Studies in the Japan Birth Cohort Consortium (JBiCC) and JECS
Source: J Epidemiol. 2023 Oct 5;33(10):498–507. doi: 10.2188/jea.JE20220076 (PMC10483100; doi:10.2188/jea.JE20220076)
Supplement: Supplementary file 1 [file je-33-498-s001.pdf]

eTable 1. Definitions of HDP and PE used in the cohorts

Hokkaido	In 2004, Japan revised the term "Toxemia of Pregnancy" to "Pregnancy Induced Hypertension," which was further revised in 2017, to "Hypertensive disorders of Pregnancy," (HDP), which is consistent with the international classification. The medical history of HDP was obtained from medical records. Since the registration period for the Hokkaido Cohort was from 2002 to 2012, HDP was defined as a combination of "Toxemia of Pregnancy" up to 2004 and "Pregnancy Induced Hypertension" after 2005. We used Japanese standard definition of HDP and subclassified the symptoms based on severity. Mild HDP (h) refers to blood pressure ≥140/90 mm Hg but <160/110 mm Hg after 20 weeks of gestation, and proteinuria (p) ≥300 mg/24 h without exceeding 2.0 g/24 h or 3 + dipstick. Severe HDP (H) refers to blood pressure ≥160/110 mmHg and proteinuria (P) exceeding 2.0 g/24 h or 3 + dipstick.[34]
TMM BirThree	An original algorithm to define HDP and preeclampsia was used. The criteria were based on those recommended by the guidelines of the American College of Obstetricians and Gynecologists (ACOG); however, hypertension (systolic blood pressure ≥140 mm Hg OR diastolic blood pressure ≥90 mm Hg) or proteinurea (≥1+) was considered positive even for cases with only one positive measurement.
Hamamatsu	HDP was reported on questionnaires filled in at birth based on medical records. HDP was defined as hypertension (systolic blood pressure ≥140 mm Hg OR diastolic blood pressure ≥90 mm Hg) occurring after 20 weeks of gestation that dissolved within 12 weeks after delivery.
BOSHI	HDP was defined as hypertension (systolic blood pressure ≥140 mm Hg OR diastolic blood pressure ≥90 mm Hg) observed more than twice on different occasions after 20 weeks of gestation and before 12 weeks after delivery, or intake of medications for hypertension.<br>Preeclampsia was defined as HDP with proteinurea, defined as any of the following: dipstick proteinurea of 1+ observed multiple times, more than one occurrence of dipstick proteinurea of 2+, proteinurea ≥27 mg/g Cre, proteinurea ≥30mg/dL.

eTable 2. Definition of smoking and not smoking during pregnancy

	Questions asked in study	Analysis 1,3		Analysis 2	
		main analysis	sensitivity analysis	main analysis	sensitivity analysis
TMM BirThree Cohort	Never smoked	0	0	0	0
	Used to smoke but quit before getting pregnant	0	0	0	0
	Used to smoke but quit after getting pregnant (before 12 weeks)	1	1	1	1
	Used to smoke but quit after getting pregnant (12 weeks or after)	1	1	2	2
	I smoke	1	1	2	2
	no answer	exclude	exclude	exclude	exclude
HBC	Never smoked.	0	0	0	0
	Never smoked after pregnancy.	1	1	1	0
	Never smoked after 12 weeks of pregnancy.	1	1	1	1
	Never smoked after 24 weeks of pregnancy.	1	1	2	2
	Little or no smoking after 24 weeks of pregnancy.	1	1	2	2
	Smoke every day.	1	1	2	2
	no answer	exclude	exclude	exclude	exclude
Hokkaido Cohort	Never smoked	0	0	0	0
	Smoked before & Did not smoke before pregnancy	0	0	0	0
	Smoked before & Smoked before pregnancy & Did not smoke during pregnancy	1	0	1	0
	Smoked before & Smoked before pregnancy & Quit during pregnancy (before 12 weeks)	1	1	1	1
	Smoked before & Smoked before pregnancy & Quit during pregnancy (12 weeks or after)	1	1	2	2
	Smoked before & Smoked before pregnancy & Smoked during pregnancy	1	1	2	2
	Missing data to categorize	exclude	exclude	exclude	exclude
BOSHI cohort	Do not smoke	0	0	0	0
	Smoker, & # of cigarettes per day before pregnancy is 0 or missing, & # of cigarettes per day is 0 or missing	0	0	0	0
	Smoker, & # of cigarettes per day before pregnancy is 1 or above, & # of cigarettes per day is 0 or missing	1	0	1	0
	Smoker, & # of cigarettes per day before pregnancy is 0 or missing, & # of cigarettes per day is 1 or above	1	1	2	2
	Smoker, & # of cigarettes per day before pregnancy is 1 or above, & number of cigarettes per day is 1 or above	1	1	2	2

For analysis 1 and 3: 0 is never smoker and those who stopped smoking before pregnancy, 1 is those who smoked during early pregnancy
For analysis 2: 0 is never smoker and those who stopped smoking before pregnancy, 1 is those who quit smoking during early pregnancy, 2 is those who smoked beyond early pregnancy

eFigure 1. Association of smoking during early pregnancy with risk of HDP and PE

A) HDP

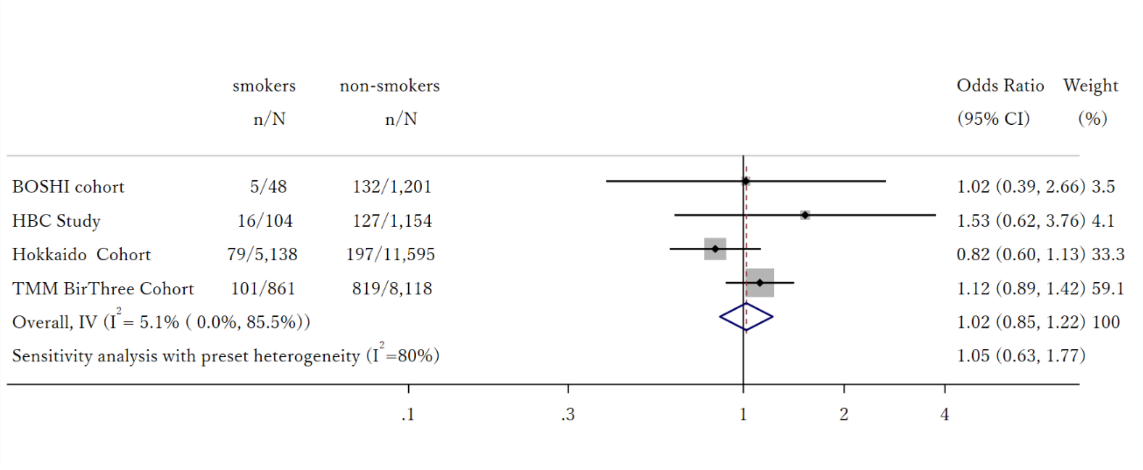

B) PE

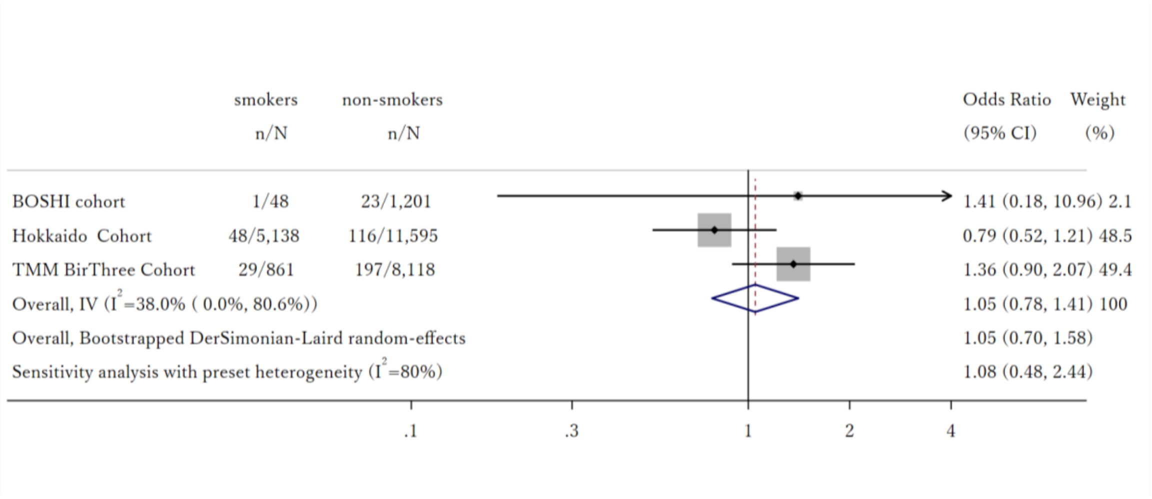

eFigure 2. Association of smoking only during early pregnancy and continuing smoking beyond pregnancy with risk of HDP and PE

A) HDP

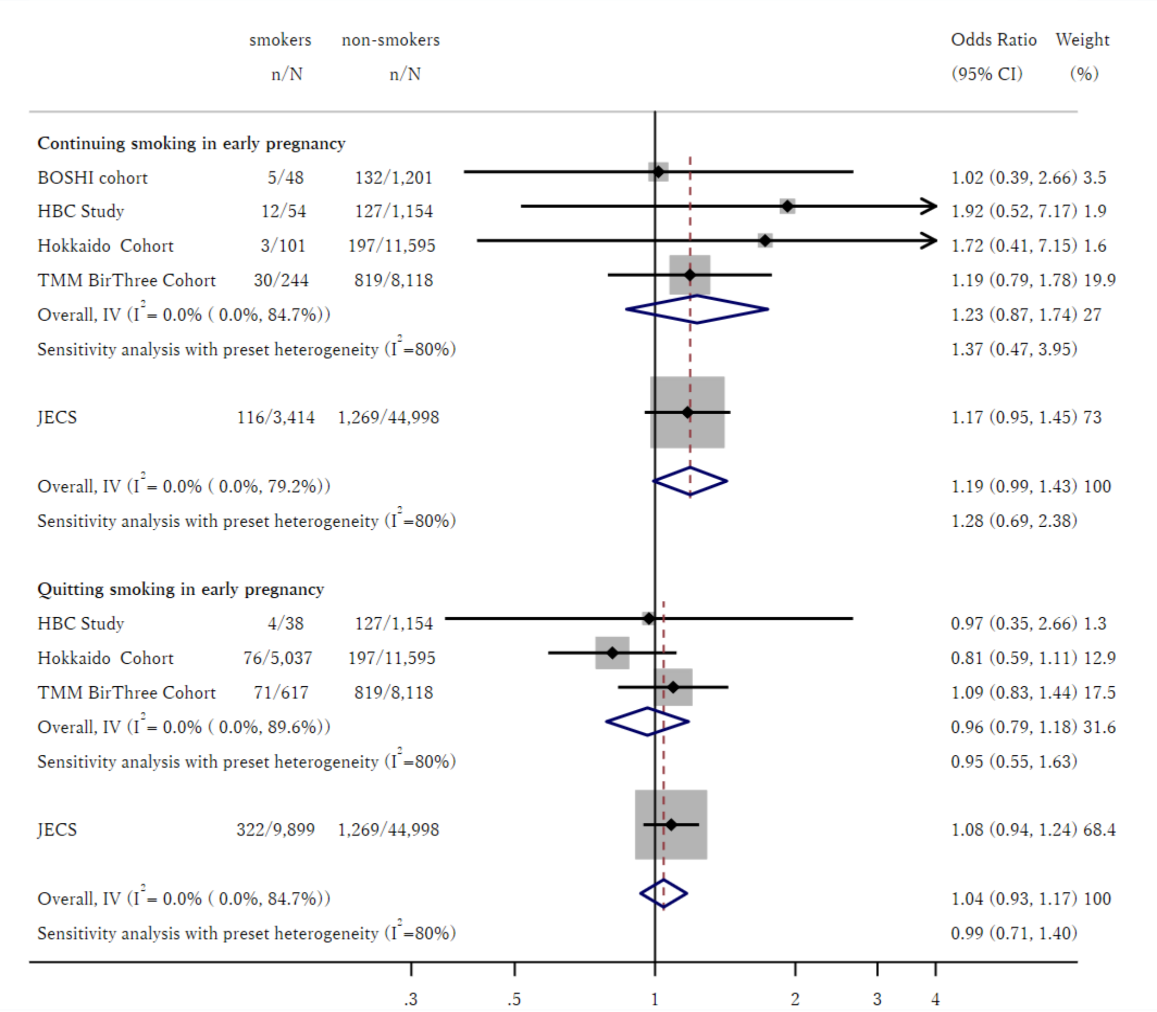

B) PE

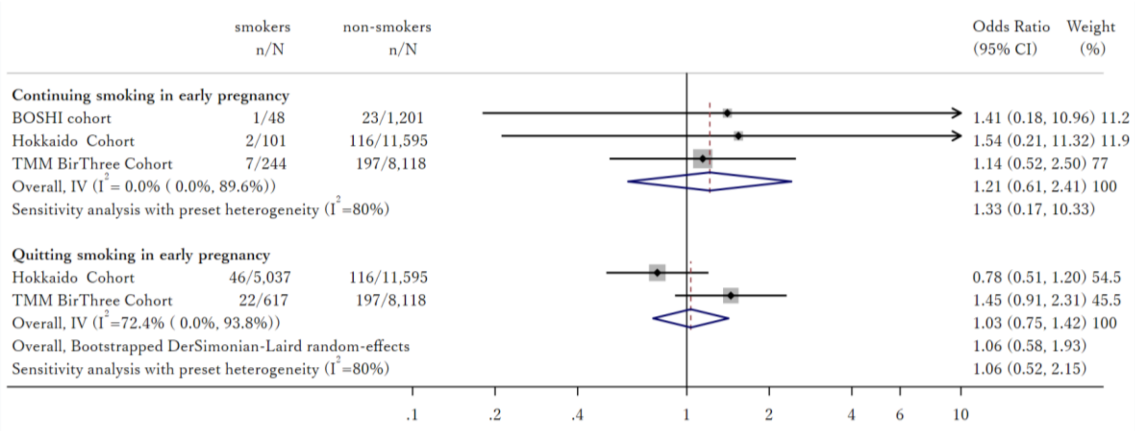
smokers n/N
non-smokers n/N
Odds Ratio (95% CI)
Weight (%)
Continuing smoking in early pregnancy
BOSHI cohort 1/48 23/1,201 1.41 (0.18, 10.96) 11.2
Hokkaido Cohort 2/101 116/11,595 1.54 (0.21, 11.32) 11.9
TMM BirThree Cohort 7/244 197/8,118 1.14 (0.52, 2.50) 77
Overall, IV (I^2 = 0.0% (0.0%, 89.6%)) 1.21 (0.61, 2.41) 100
Sensitivity analysis with preset heterogeneity (I^2=80%) 1.33 (0.17, 10.33)
Quitting smoking in early pregnancy
Hokkaido Cohort 46/5,037 116/11,595 0.78 (0.51, 1.20) 54.5
TMM BirThree Cohort 22/617 197/8,118 1.45 (0.91, 2.31) 45.5
Overall, IV (I^2=72.4% (0.0%, 93.8%)) 1.03 (0.75, 1.42) 100
Overall, Bootstrapped DerSimonian-Laird random-effects 1.06 (0.58, 1.93)
Sensitivity analysis with preset heterogeneity (I^2=80%) 1.06 (0.52, 2.15)
.1
.2
.4
1
2
4
6
10

eFigure 3. Association of volume of smoking during early pregnancy with risk of HDP and PE

A) HDP

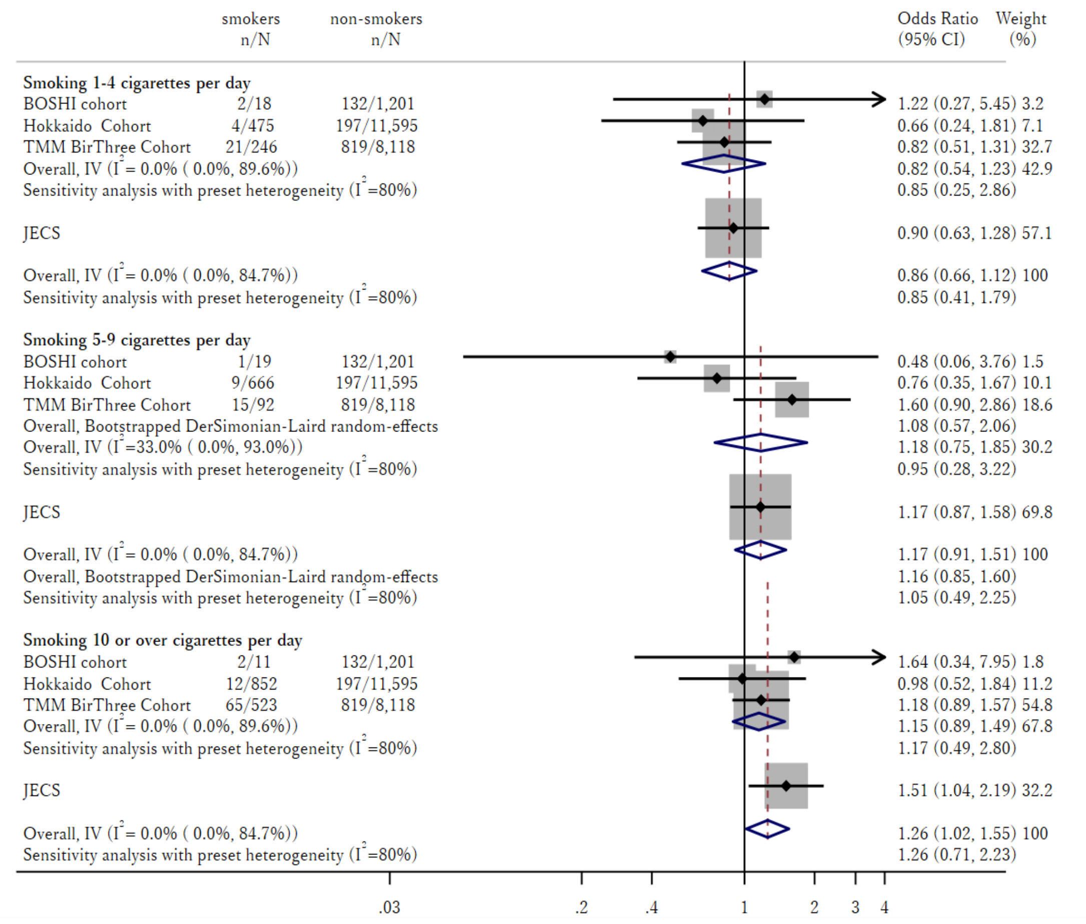

B) PE

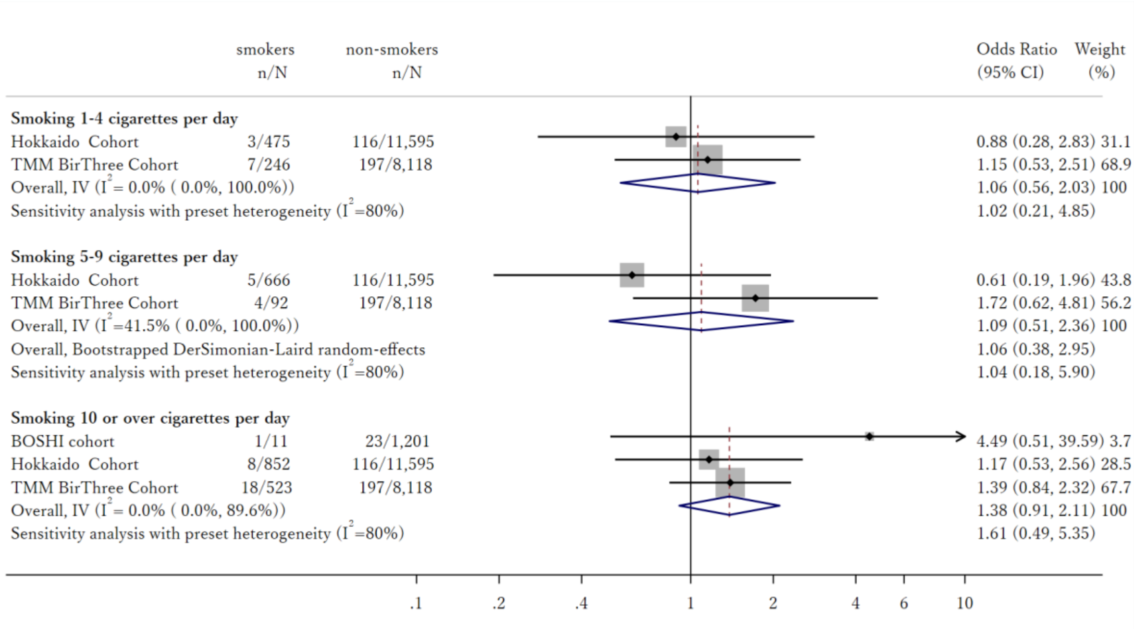
smokers n/N
non-smokers n/N
Odds Ratio (95% CI)
Weight (%)
Smoking 1-4 cigarettes per day
Hokkaido Cohort 3/475 116/11,595 0.88 (0.28, 2.83) 31.1
TMM BirThree Cohort 7/246 197/8,118 1.15 (0.53, 2.51) 68.9
Overall, IV (I2 = 0.0% (0.0%, 100.0%)) 1.06 (0.56, 2.03) 100
Sensitivity analysis with preset heterogeneity (I2=80%) 1.02 (0.21, 4.85)
Smoking 5-9 cigarettes per day
Hokkaido Cohort 5/666 116/11,595 0.61 (0.19, 1.96) 43.8
TMM BirThree Cohort 4/92 197/8,118 1.72 (0.62, 4.81) 56.2
Overall, IV (I2=41.5% (0.0%, 100.0%)) 1.09 (0.51, 2.36) 100
Overall, Bootstrapped DerSimonian-Laird random-effects 1.06 (0.38, 2.95)
Sensitivity analysis with preset heterogeneity (I2=80%) 1.04 (0.18, 5.90)
Smoking 10 or over cigarettes per day
BOSHI cohort 1/11 23/1,201 4.49 (0.51, 39.59) 3.7
Hokkaido Cohort 8/852 116/11,595 1.17 (0.53, 2.56) 28.5
TMM BirThree Cohort 18/523 197/8,118 1.39 (0.84, 2.32) 67.7
Overall, IV (I2 = 0.0% (0.0%, 89.6%)) 1.38 (0.91, 2.11) 100
Sensitivity analysis with preset heterogeneity (I2=80%) 1.61 (0.49, 5.35)
.1 .2 .4 1 2 4 6 10

eFigure 4. Influence analysis plot: meta-analysis estimates, given named study is omitted

A) Smoking during early pregnancy and HDP

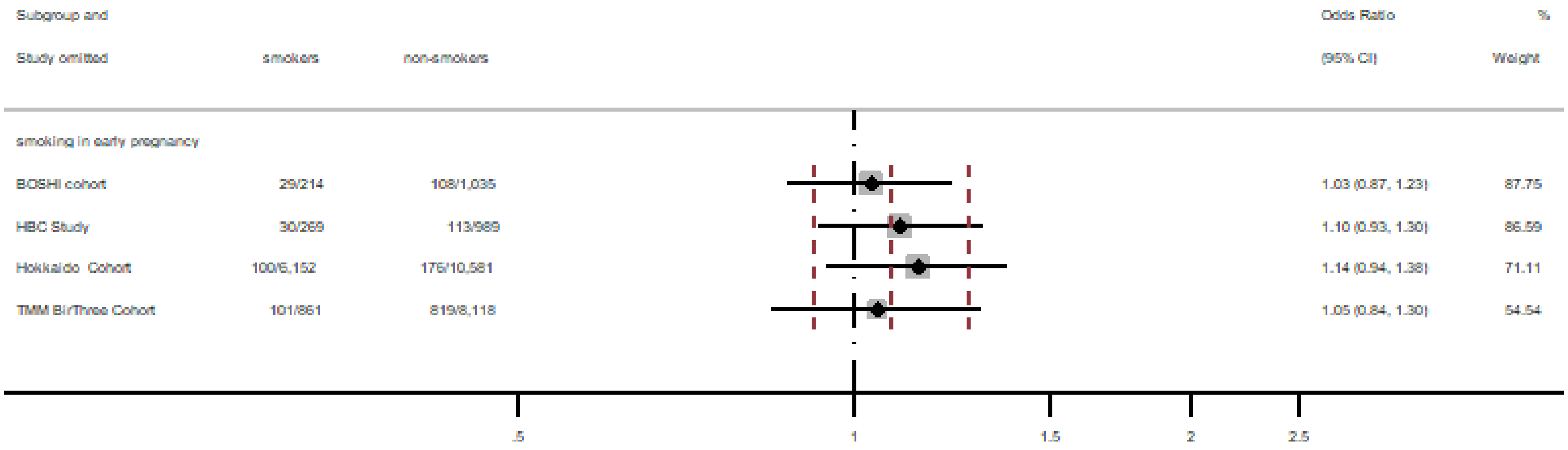

B) Quitting smoking and continuing smoking beyond early pregnancy and HDP

Subgroup and Study omitted	smokers	non-smokers	Odds Ratio (95% CI)	% Weight
continuing smoking in early pregnancy				
BOSHI cohort	5/48	108/1,035	1.27 (0.87, 1.84)	87.11
HBC Study	12/66	113/989	1.21 (0.84, 1.73)	92.31
Hokkaido Cohort	3/101	176/10,581	1.22 (0.85, 1.74)	94.06
TMM BirThree Cohort	30/244	819/8,118	1.41 (0.72, 2.78)	26.52
stopping smoking in early pregnancy				
BOSHI cohort	24/166	108/1,035	0.95 (0.80, 1.14)	88.34
HBC Study	18/203	113/989	1.08 (0.90, 1.30)	82.02
Hokkaido Cohort	97/6,051	176/10,581	1.05 (0.85, 1.29)	67.95
TMM BirThree Cohort	71/617	819/8,118	0.96 (0.78, 1.19)	61.69

Heterogeneity between groups: p = 0.289

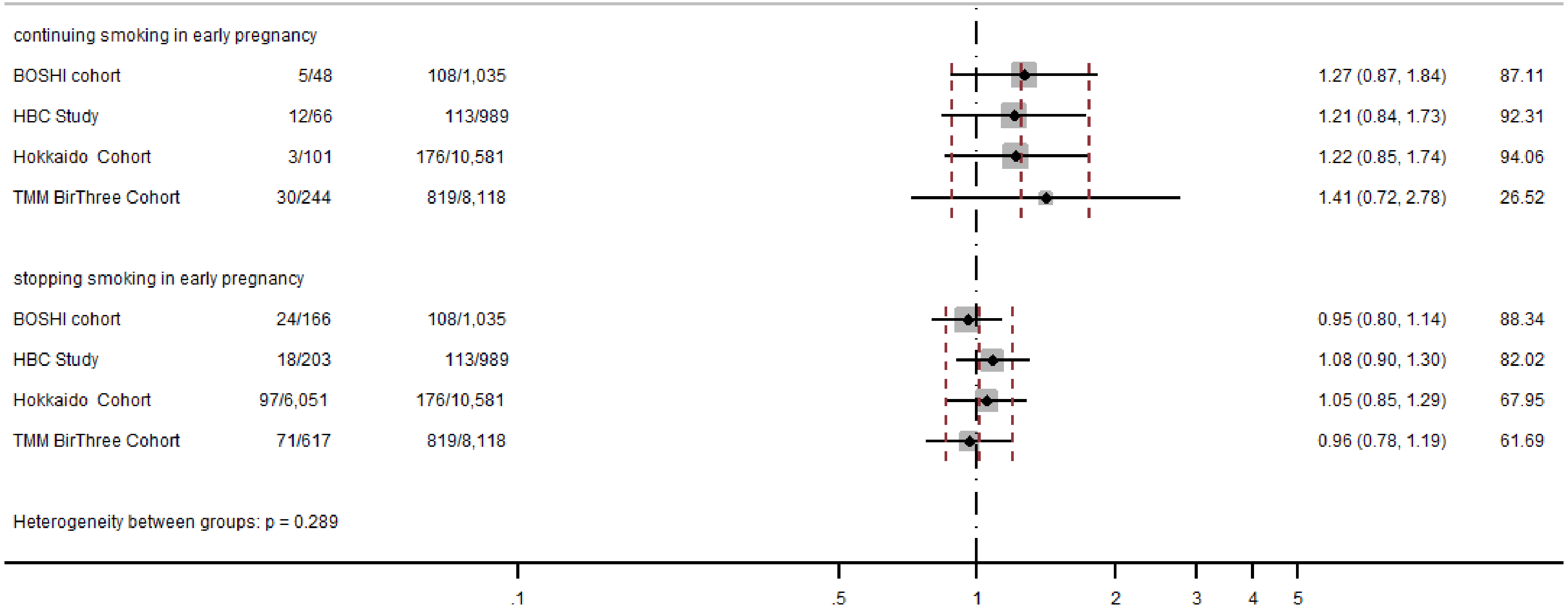

Morisaki et al. Association between smoking and hypertension in pregnancy among Japanese women: a meta-analysis of birth cohort studies in the Japan Birth Cohort Consortium (JBiCC) and JECS. *Journal of Epidemiology*

C) Volume of smoking during early pregnancy and HDP

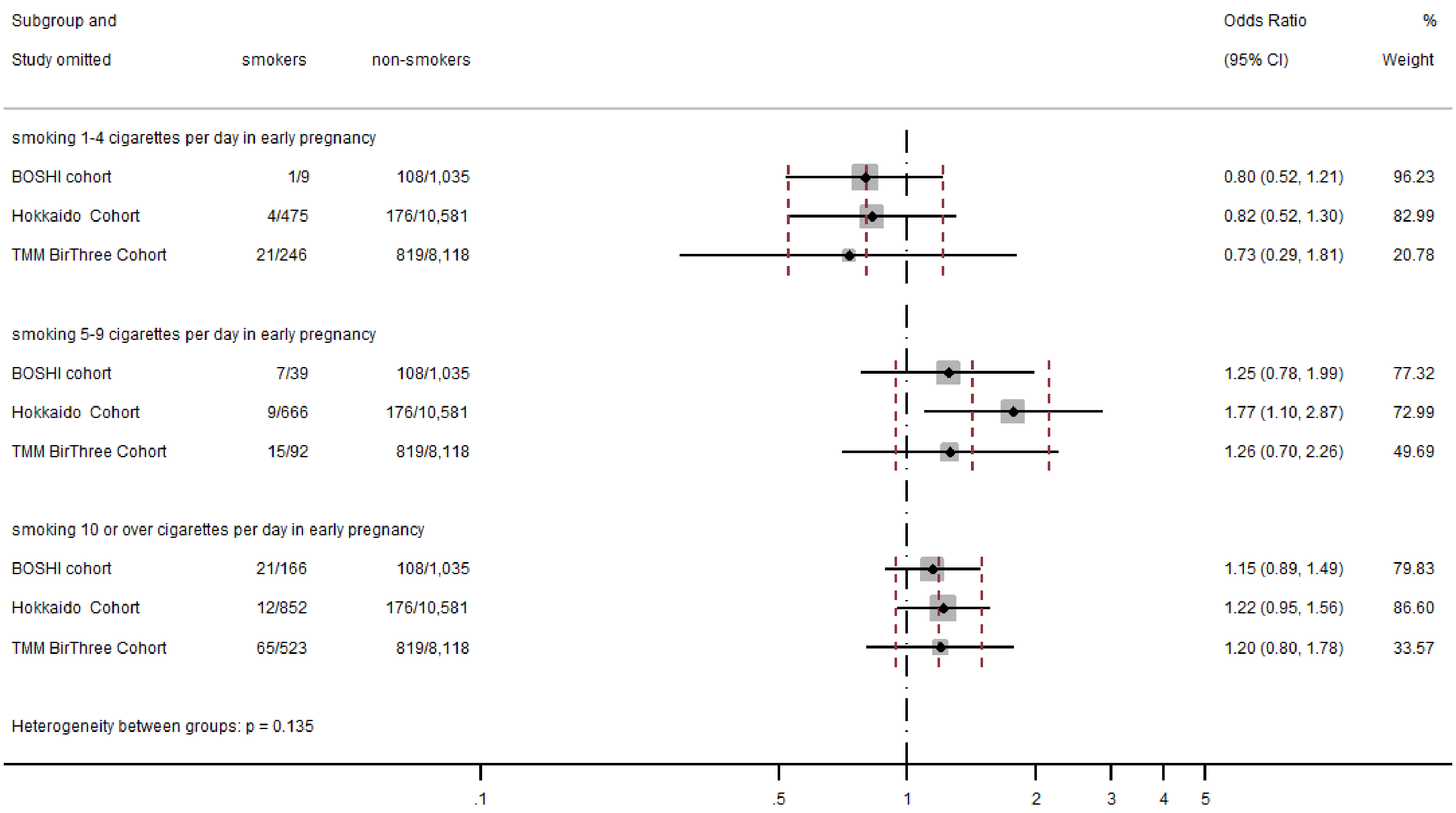

Morisaki et al. Association between smoking and hypertension in pregnancy among Japanese women: a meta-analysis of birth cohort studies in the Japan Birth Cohort Consortium (JBiCC) and JECS. *Journal of Epidemiology*
